# A novel lncRNA RP11-544M22.13 enhances glycolysis-induced cisplatin resistance in non-small cell lung cancer

**DOI:** 10.1038/s41420-025-02873-3

**Published:** 2025-11-27

**Authors:** Jun Xiong, Haiyue Zhang, Zhou Pan, Yixuan Wang, Hui Chen, Mei Yang

**Affiliations:** 1 Department of Emergency, Renmin Hospital of Wuhan University, Wuhan, Hubei Province China; 2 Department of Pulmonary and Critical Care Medicine, Renmin Hospital of Wuhan University, Wuhan, Hubei Province China; 3 Department of Cardiology, Renmin Hospital of Wuhan University, Wuhan, Hubei Province China

**Keywords:** Non-small-cell lung cancer, Predictive markers, Mechanisms of disease

## Abstract

LncRNAs and glycolysis play integral roles in the advancement and development of different types of cancer, such as non-small cell lung cancer (NSCLC). This particular study aims to delve into the specific function and potential mechanism of RP11-544M22.13, a newly identified lncRNA, in NSCLC. We utilized qRT-PCR to measure RP11-544M22.13, miR-1291, and SLC2A1 expression levels. The MTT assay assessed NSCLC cell chemoresistance. RP11-544M22.13 was highly elevated in diamminedichloroplatinum (DDP)-resistant NSCLC tissues and cells, promoting both DDP resistance and malignant behavior. Its mechanism involved targeting SLC2A1, suppressing miR-1291, and subsequently upregulating SLC2A1 expression via direct interaction with SLC2A1 mRNA. RP11-544M22.13 acts as a miR-1291 sponge, boosting SLC2A1 expression and promoting DDP resistance and malignancy in NSCLC cells. This presents a potential treatment target for NSCLC and aids in developing small-molecule drugs for combined chemotherapy in late-stage, chemoresistant NSCLC patients.

## Introduction

NSCLC, a prevalent solid tumor with high mortality rates, poses a significant global health challenge. Diamminedichloroplatinum (DDP) is a commonly employed chemotherapy agent for NSCLC treatment [1, 2]. Unfortunately, many NSCLC patients develop resistance to DDP, which in turn leads to tumor recurrence and metastasis [3]. Consequently, there is an immediate need to comprehend the mechanisms underlying DDP resistance in NSCLC cells to identify novel therapeutic targets.

Non-coding RNAs (ncRNAs), such as lncRNAs, circRNAs, and miRNAs, play a crucial role in regulating cancer drug resistance, including in NSCLC [4]. For example, lncRNA SNHG1 contributes to the cisplatin resistance and progression of NSCLC via miR-330-5p/DCLK1 axis [5]. The Warburg effect, or aerobic glycolysis, is a key metabolic behavior in tumor progression where cells convert glucose to lactate even with oxygen present [6]. LncRNAs contribute to NSCLC progression through glycolysis [7]. Glycolysis is closely linked to chemoresistance [8]. However, the role of lncRNA-mediated glycolysis in NSCLC chemosensitivity is not well understood.

In this research, we discovered a new lncRNA called RP11-544M22.13 (ENST00000289779.3) that is highly expressed in NSCLC tissues and strongly linked to DDP resistance. Through transcriptomic and metabolomic analysis, we found that RP11-544M22.13 controls the glycolysis pathway in drug-resistant NSCLC cells. It achieves this by acting as a sponge for miR-1291, which regulates the expression of SLC2A1, a key gene in glycolysis. This mechanism enhances DDP resistance in NSCLC by promoting glycolysis through SLC2A1. Furthermore, we identified the transcription factor USF1 as the cause of RP11-544M22.13’s high expression in NSCLC.

## Results

### The highly expressed lncRNA RP11-544M22.13 is associated with DDP resistance and poor prognosis in NSCLC

**Fig. 1:**
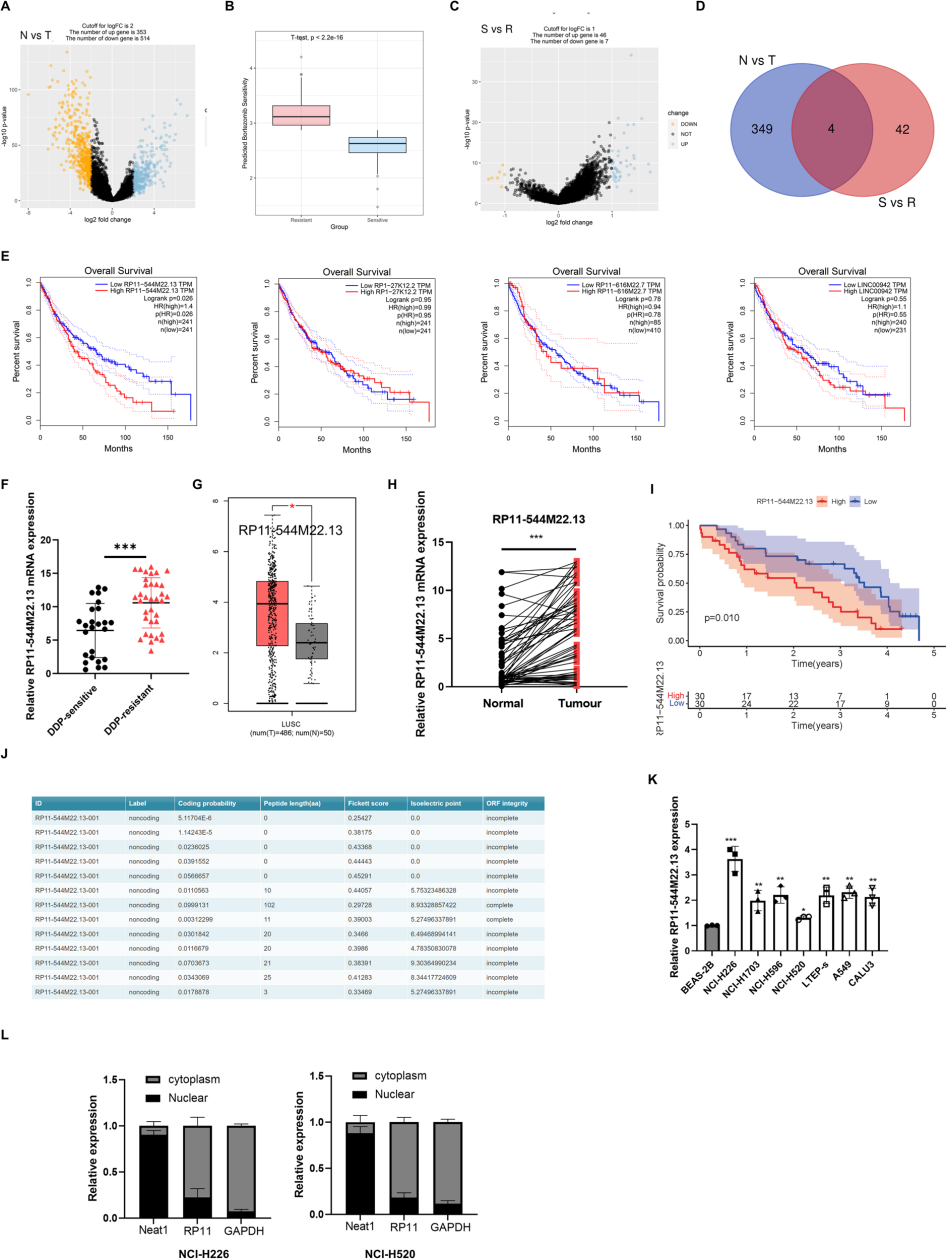
RP11-544M22.13 is associated with DDP resistance in NSCLC, and its expression is significantly upregulated in NSCLC. **A** Differential expression analysis of lncRNAs between NSCLC and adjacent normal tissues based on TCGA data. **B** Drug sensitivity analysis. **C** Analysis of differential expression of lncRNAs between cisplatin-resistant and sensitive groups based on TCGA data. **D** Venn diagram illustrating lncRNAs upregulated in both NSCLC and cisplatin-resistant NSCLC groups. **E** Survival curves of NSCLC patients stratified by different expression groups. **F** The expression of RP11-544M22.13 in DDP-resistant/non-resistant NSCLC tissues. **G** The expression of RP11-544M22.13 in cancer and para-cancerous tissues in the TCGA database. **H** Expression of RP11-544M22.13 in cancer and paracancerous tissues in NSCLC samples from Wuhan University Renmin Hospital. **I** Based on RP11-544M22.13 expression Survival curves of grouped NSCLC patients from Renmin Hospital of Wuhan University. **J** Coding properties of RP11-544M22.13 transcript predicted by CPT2. **K** RP11-544M22.13 expression in NSCLC cell lines and normal lung epithelial cell lines. **L** Nuclear-cytoplasmic separation experiment detects the subcellular localization of RP11-544M22.13 in NSCLC cell lines.

First, transcriptome expression data from 486 NSCLC samples and 50 normal lung tissues were collected and analyzed using the TCGA database. Differential analysis revealed that 353 lncRNAs were significantly upregulated in NSCLC compared to normal lung tissues (Fig. 1A). Subsequently, drug sensitivity analysis determined the IC50 values for cisplatin treatment in the 486 NSCLC samples. Based on these values, NSCLC samples were categorized into drug-resistant (Resistant, n = 243) and drug-sensitive (Sensitive, n = 243) groups, with a significant difference in IC50 values between the two groups confirmed by T-test (Fig. 1B). Differential analysis between these groups identified 46 lncRNAs that were significantly upregulated in the cisplatin-resistant group compared to the sensitive group (Fig. 1C). Intersection analysis of lncRNAs upregulated in NSCLC and those associated with cisplatin resistance identified four lncRNAs: RP11-544M22.13, RP1-27K12.2, RP11-616M22.7, and LINC00942 (Fig. 1D). Further survival analysis indicated that high expression of RP11-544M22.13 was associated with poorer prognosis in NSCLC patients, while the remaining three lncRNAs showed no significant impact on NSCLC prognosis (Fig. 1E). In summary, RP11-544M22.13 was upregulated in NSCLC, suggesting it may act as a pro-carcinogenic factor. Its upregulation in cisplatin-resistant NSCLC implies a role in drug resistance, and its association with poor prognosis suggests RP11-544M22.13 could serve as a potential biomarker for monitoring NSCLC patient outcomes. Consequently, further research will focus on RP11-544M22.13 to explore its implications in NSCLC prognosis and cisplatin resistance.

We collected tissues from 60 pairs of NSCLC patients and their matched adjacent tissues, among which 25 cases showed DDP sensitivity, while 35 cases showed DDP resistance. Through qPCR, we found that a novel lncRNA RP11-544M22.13 was significantly upregulated in DDP-resistant NSCLC tissues (Fig. 1F). Additionally, analysis of TCGA transcriptome and prognosis data revealed that RP11-544M22.13 was highly expressed in NSCLC and correlated with poor prognosis (Fig. 1G–I). Our findings were confirmed by the 60 pairs of NSCLC patient tissues. Furthermore, we found a close correlation between the high expression of RP11-544M22.13 and tumor size and lymph node metastasis in NSCLC (Supplementary Table S4). We identified the non-coding nature of RP11-544M22.13 through CPT2 analysis (Fig. 1J). The expression of RP11-544M22.13 in NSCLC cell lines was significantly higher compared to the control normal lung cell line BEAS-2B, with the highest expression in NCI-H226 and the lowest expression in NCI-H520 (Fig. 1K). Additionally, nuclear-cytoplasmic fractionation experiments showed that RP11-544M22.13 was mainly expressed in the cytoplasm (Fig. 1L).

### lncRNA RP11-544M22.13 promotes the DDP resistance of NSCLC

**Fig. 2:**
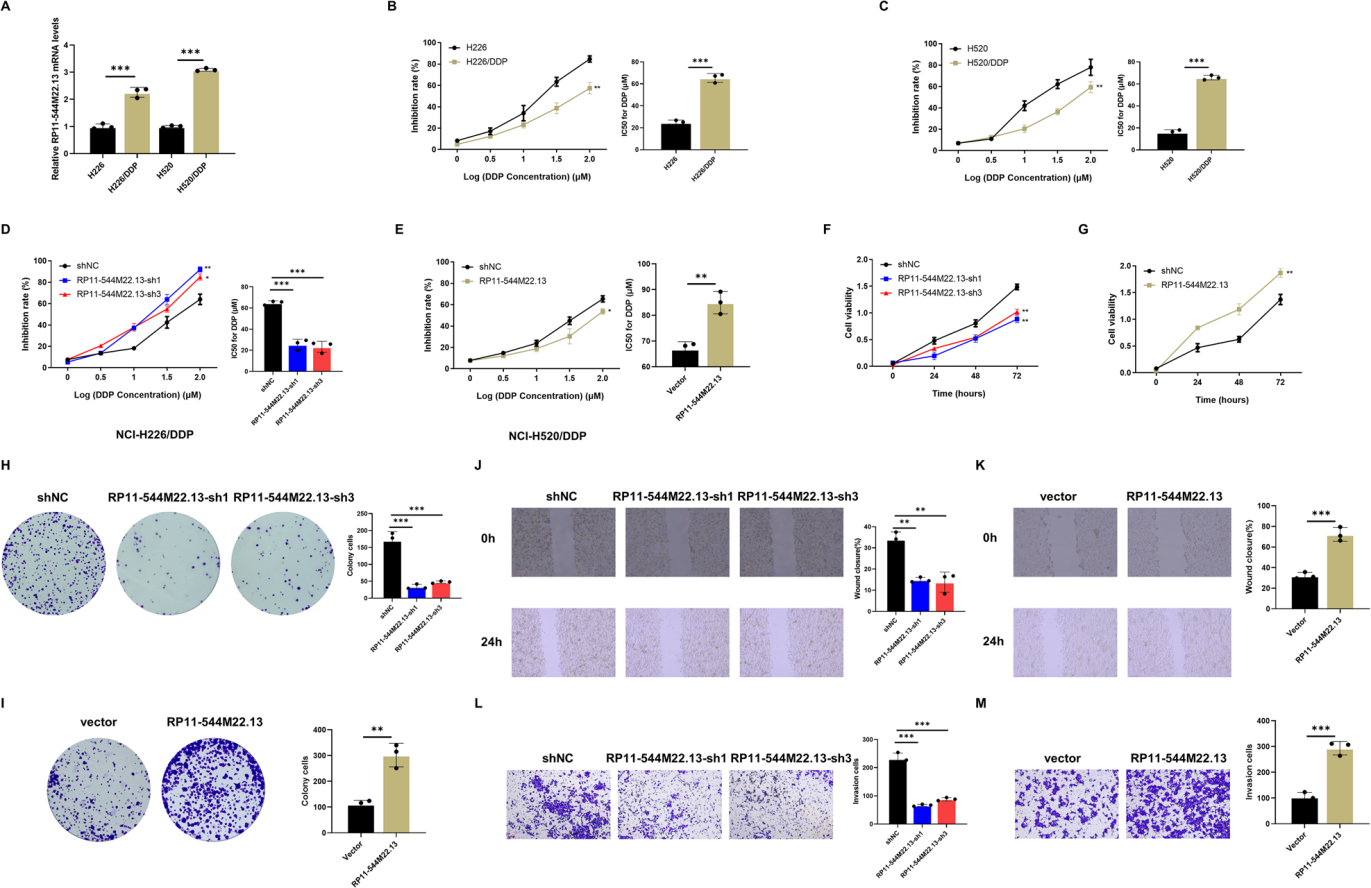
RP11-544M22.13 Promotes DDP resistance and oncological behavior in NSCLC. All NSCLC cells were treated with 1 μg/ml DDP. **A** Expression of RP11-544M22.13 in DDP-resistant NSCLC cell lines. **B**, **C** Construction of DDP-resistant NSCLC cell lines. **D** Detection of DDP sensitivity of RP11-544M22.13 knockdown NSCLC cells (**E**) Detection of DDP sensitivity of RP11-544M22.13 overexpression NSCLC cells. **F** Detection of the proliferation ability of RP11-544M22.13 knockdown NSCLC cells by CCK8 assay. **G** Detection of the proliferation ability of RP11-544M22.13 overexpression NSCLC cells by CCK8 assay. **H** The proliferation ability of RP11-544M22.13 knockdown NSCLC cells was detected by colony formation assay. **I** The proliferation ability of RP11-544M22.13 overexpressing NSCLC cells was detected by colony formation assay. **J** The migration ability of RP11-544M22.13 knockdown NSCLC cells was detected by scratch assay. **K** The migration ability of RP11-544M22.13 overexpression NSCLC cells was detected by scratch assay. **L** By Transwell Experiment to detect the invasive ability of RP11-544M22.13 knockdown NSCLC cells. **M** Transwell assay to detect the invasive ability of RP11-544M22.13 overexpressing NSCLC cells.

In order to investigate the impact of RP11-544M22.13 on DDP resistance in NSCLC, we constructed the DDP-resistant cell lines NCI-H226/DDP and NCI-H520/DDP (Fig. 2A). Knockdown models were established using NCI-H226/DDP, while overexpression models were generated using NCI-H520/DDP (Fig. 2B, C). We found that knockdown of RP11-544M22.13 significantly enhanced the sensitivity of NSCLC to DDP (Fig. 2D). Additionally, CCK8 and colony-forming assays showed that knockdown of RP11-544M22.13 significantly reduced the proliferative capacity of NSCLC (Fig. 2F, H). Transwell and scratch assays indicated that knockdown of RP11-544M22.13 greatly reduced the invasive and migratory abilities of NSCLC (Fig. 2J, L). Conversely, overexpression of RP11-544M22.13 yielded opposite results. These experiments suggest that lncRNA RP11-544M22.13 promotes the DDP resistance and oncogenic behavior of NSCLC (Fig. 2E, G, I, K, M).

### lncRNA RP11-544M22.13 promotes glycolysis in DPP-resistant NSCLC

**Fig. 3:**
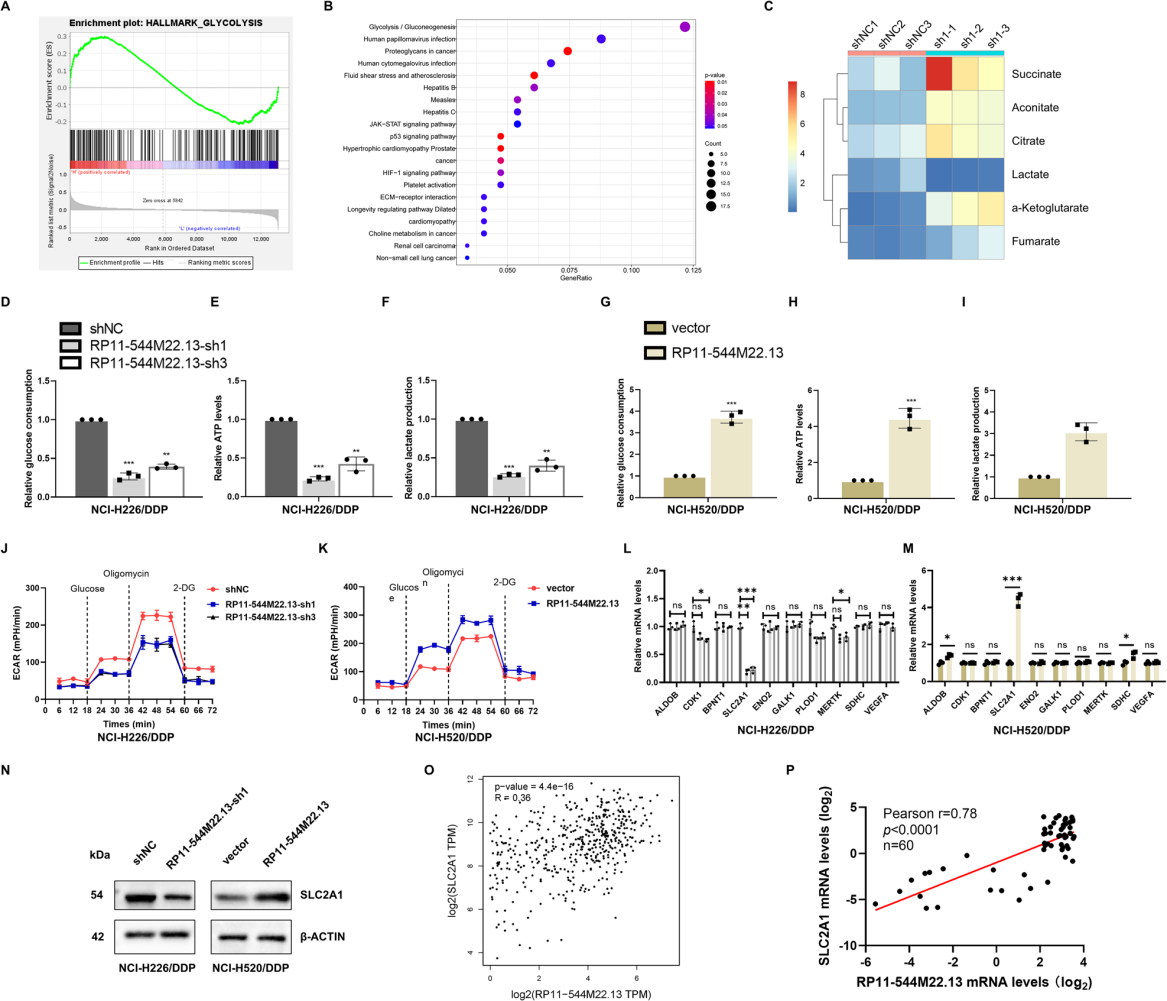
RP11-544M22.13 promotes the glycolytic ability of NSCLC. All NSCLC cells were treated with 1 μg/ml DDP. **A** GESA enrichment analysis by transcriptomics. **B** KEGG enrichment analysis of differential genes by transcriptomics. **C** Metabolomics analysis by knockdown of RP11-544M22.13. **D**–**F** Knockdown of RP11- 544M22.13 was used to detect glucose production, ATP levels and lactate production. **G**–**I** Overexpression of RP11-544M22.13 was used to detect glucose production, ATP levels and lactate production. **J**, **K** Changes of ECAR rate in NSCLC cells. **L** Knockdown RP11-544M22.13 for qRT-PCR detection of key glycolysis genes. **M** Overexpression of RP11-544M22.13 for qRT-PCR detection of key glycolysis genes. **N** Intervention of RP11- 544M22.13 expression, Western blot analysis of the key glycolysis gene SLC2A1. **O** Correlation analysis of RP11-544M22.13 and SLC2A1 in the TCGA database. **P** Correlation analysis of RP11-544M22.13 and SLC2A1 in NSCLC samples from Wuhan University Renmin Hospital.

In order to clarify the specific mechanism of lncRNA RP11-544M22.13 regulating the DDP resistance of NSCLC, we performed transcriptomics on RP11-544M22.13 knockdown NCI-H226/DDP cells. The results of enrichment analysis showed that RP11-544M22.13 significantly regulated the glycolysis pathway in NCI-H226/DDP cells (Fig. 3A, B). We confirmed this by metabolomics, where knockdown of RP11-544M22.13 significantly increased the content of gluconeogenesis products such as Succinate, while decreased the content of glycolysis products lactate (Fig. 3C). Through biochemical detection, we found that knockdown of RP11-544M22.13 significantly reduced the glucose uptake, ATP production and lactic acid production of NSCLC cells (Fig. 3D–F). And RP11-544M22.13 knockdown significantly reduced the ECAR level in NSCLC cells (Fig. 3J). The overexpression of RP11-544M22.13 showed the above opposite results (Fig. 3G–I, K). Next, we detected ten glycolytic differential genes that RP11-544M22.13 may regulate as shown by transcriptomics, and we found that only SLC2A1 was knocked down and overexpressed by knocked down and overexpressed RP11-544M22 (Fig. 3L, M). Western-blot analysis showed that RP11-544M22.13 knockdown significantly reduced the protein level of SLC2A1, while RP11-544M22.13 overexpression increased the protein level of SLC2A1 (Fig. 3N). In addition, correlation analyzes of the NSCLC cohorts of TCGA and Renmin Hospital of Wuhan University both showed a strong correlation of RP11-544M22.13 and SLC2A1 in NSCLC tissues(Fig. 3O, P).

### LncRNA RP11-544M22.13 promotes glycolysis and DDP resistance in NSCLC through SLC2A1 in vitro and in vivo

**Fig. 4:**
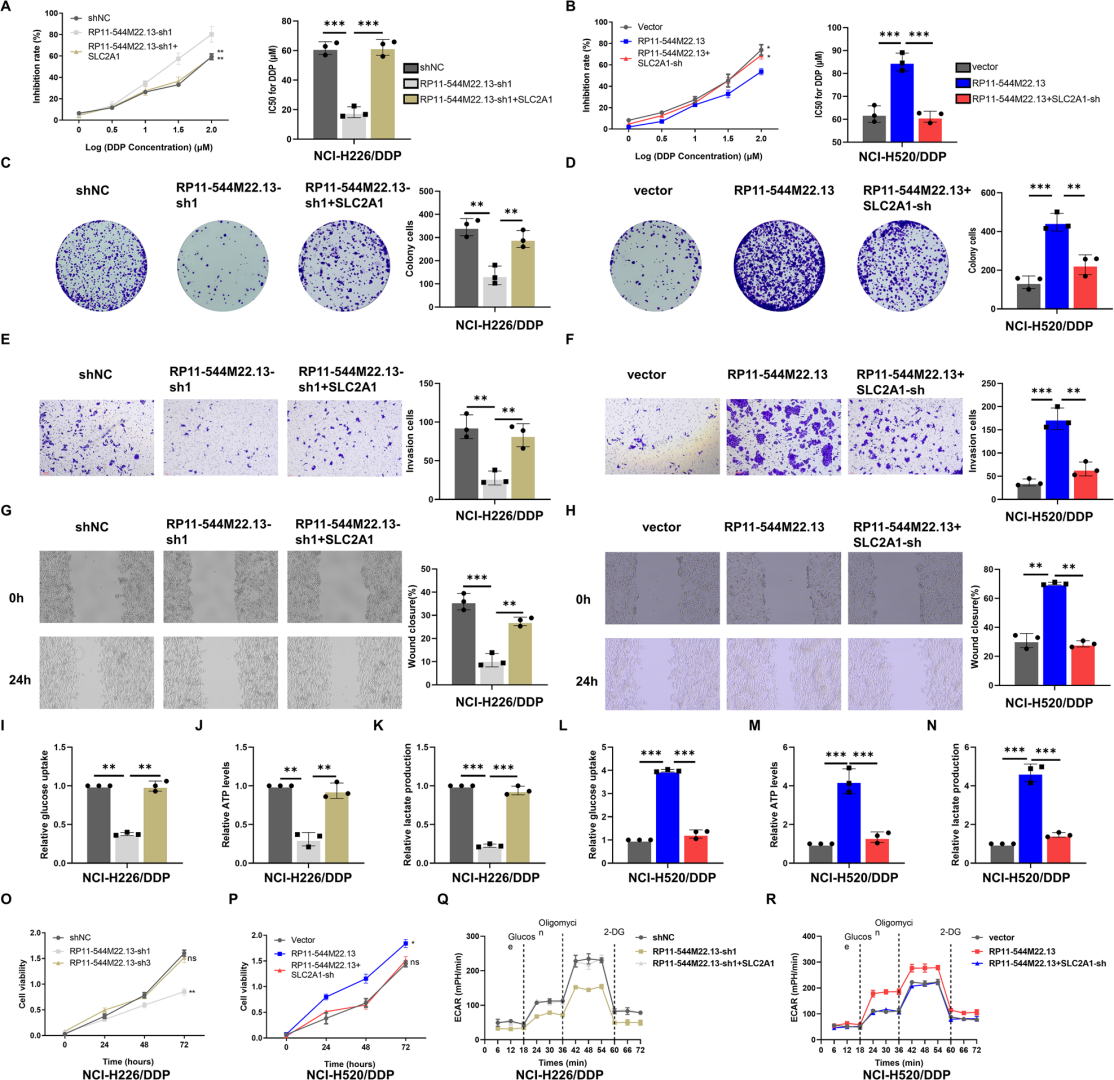
RP11-544M22.13 promotes NSCLC progression and glycolysis through SLC2A1. All NSCLC cells were treated with 1 μg/ml DDP. **A** Effect of SLC2A1 overexpression on DDP resistance of RP11-544M22.13 knockdown NSCLC cells. **B** Effect of SLC2A1 knockdown on DDP resistance of RP11-544M22.13 overexpression NSCLC cells. **C** SLC2A1 overexpression Effect on the proliferation ability of RP11-544M22.13 knockdown NSCLC cells. **D** Effect of SLC2A1 knockdown on the proliferation ability of RP11-544M22.13 overexpression NSCLC cells. **E** Effect of SLC2A1 overexpression and RP11-544M22.13 knockdown on the invasion ability of NSCLC cells. **F** Effect of SLC2A1 knockdown on the invasion ability of NSCLC cells with RP11-544M22.13 overexpression. **G** Effect of SLC2A1 overexpression on the migration ability of RP11-544M22.13 knockdown NSCLC cells. **H** Effect of SLC2A1 knockdown on the migration ability of RP11-544M22.13 overexpression NSCLC cells. **I**–**K** Effect of SLC2A1 overexpression on RP11-544M22.13 knockdown NSCLC cells glucose production, ATP levels and lactate production. **L**–**N** Effect of SLC2A1 knockdown on glucose production, ATP levels and lactate production of RP11-544M22.13 overexpression NSCLC cells. **O** CCK8 detection of SLC2A1 overexpression on RP11-544M22.13 knockdown NSCLC cells. **P** CCK8 detection of SLC2A1 knockdown on RP11-544M22.13 overexpressing NSCLC cells. **Q** Detection of ECAR rate in SLC2A1 overexpression in RP11-544M22.13 knockdown NSCLC cells. **R** SLC2A1 knockdown in RP11-544M22.13 overexpression NSCLC cells. Detection of ECAR rate of cells.

Most lncRNAs located in the cytoplasm regulate the expression of downstream target genes through the ceRNA mechanism [5, 9, 10]. In order to verify whether RP11-544M22.13 promotes glycolysis and DDP resistance in NSCLC through SLC2A1, we performed functional recovery experiments. We found that overexpression of SLC2A1 reduced the DDP sensitivity of RP11-544M22.13 knockdown-enhanced NSCLC cells (Fig. 4A) and the overexpression of SLC2A1 rescued the decrease of proliferation, invasion and migration ability of NSCLC cells induced by RP11-544M22.13 knockdown (Fig. 4C, E, G). In terms of metabolism, overexpression of SLC2A1 rescued the reduction of the glucose uptake, ATP production, lactic acid production and ECAR level in NSCLC cells induced by RP11-544M22.13 knockdown (Fig. 4I–K, O, Q). Knockdown of SLC2A1 led to the above opposite results (Fig. 4B, D, F, H, L–N, P, R). The above results suggest that RP11-544M22.13 promotes glycolysis and DDP resistance in NSCLC through SLC2A1 in vitro.

**Fig. 5:**
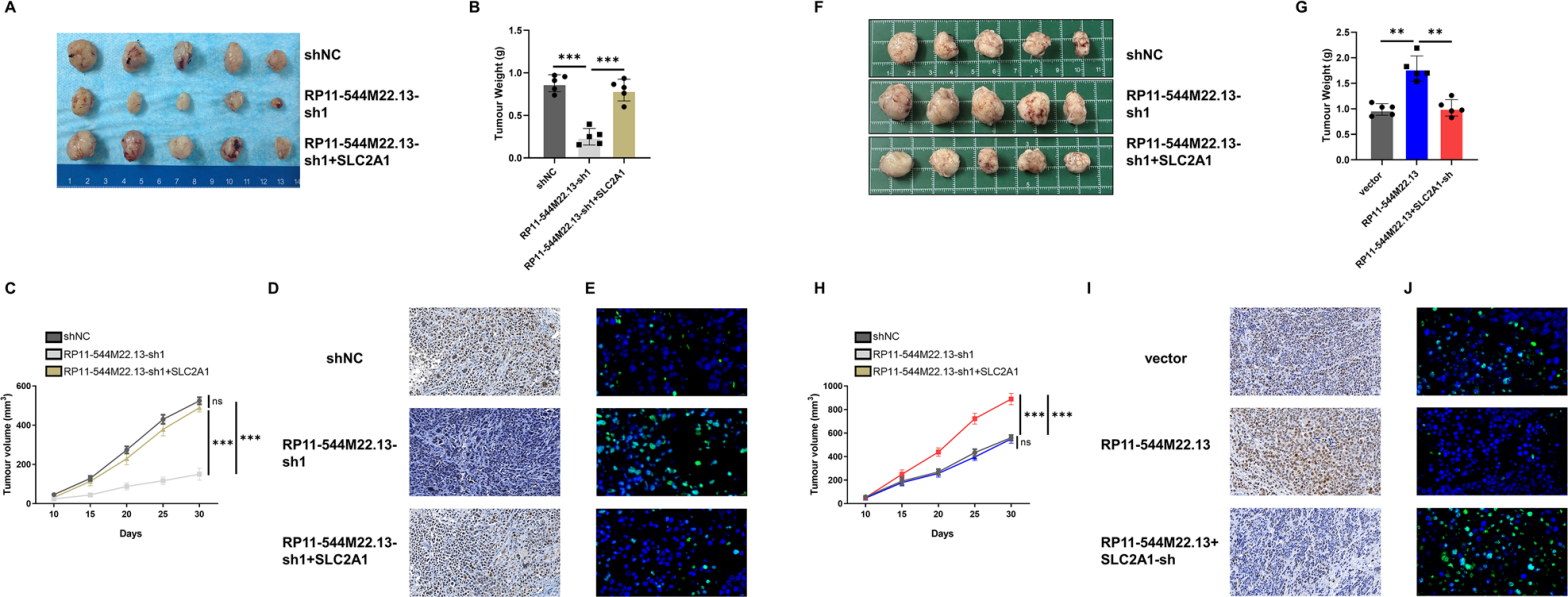
The RP11-544M22.13/SLC2A1 axis promotes the DDP resistance of NSCLC in vivo. All NSCLC cells were treated with 1 μg/ml DDP. **A** RP11-544M22.13 knockdown and SLC2A1 overexpression NCI-H226 cells were subcutaneously implanted into the left axilla of BALB/c-nu mice for tumor formation experiment. **B** Measure the size of subcutaneous tumors. **C** Measure the growth rate of subcutaneous tumors. **D** KI-67 staining demonstrates the proliferative capacity of tumor cells, 400x magnification. **E** TUNEL staining reveals the apoptotic level of tumor cells, 400× magnification. **F** RP11-544M22.13 overexpression and SLC2A1 knockdown NCI-H520 cells were subcutaneously implanted into the left axilla of BALB/c-nu mice for tumor formation experiment. **G** Measure the size of subcutaneous tumors. **H** Measure the growth rate of subcutaneous tumors. **I** KI-67 staining demonstrates the proliferative capacity of tumor cells, 400x magnification. **J** TUNEL staining reveals the apoptotic level of tumor cells, 400× magnification.

We then constructed a Xenograft model. After subcutaneous tumor formation, DDP (5 mg/kg per day) was injected intraperitoneally into mice. We found that knockdown of RP11-544M22.13 inhibited the proliferation rate and volume of subcutaneous tumors, which were rescued by overexpression of SLC2A1 (Fig. 5A–C). In addition, knockdown of RP11-544M22.13 significantly reduced the levels of ki67 in tumor cells, which was rescued by overexpression of SLC2A1 (Fig. 5D). Tunel staining demonstrated that knockdown of RP11-544M22.13 significantly increased the apoptosis levels of tumor cells, whereas overexpression of SLC2A1 decreased it (Fig. 5E). Further experiments found that overexpression of RP11-544M22.13 promoted the proliferation rate and volume of subcutaneous tumors, while knockdown of SLC2A1 rescued this phenomenon (Fig. 5F–H). In addition, overexpression of RP11-544M22.13 significantly increased the level of KI-67 in tumor cells, while knockdown of SLC2A1 rescued this level (Fig. 5D). Tunel staining showed that overexpression of RP11-544M22.13 significantly reduced the apoptosis level of tumor cells, while knockdown of SLC2A1 increased the apoptosis level of tumor cells (Fig. 5E).

### LncRNA RP11-544M22.13 regulates the expression of SLC2A1 through sponging miR-1291

**Fig. 6:**
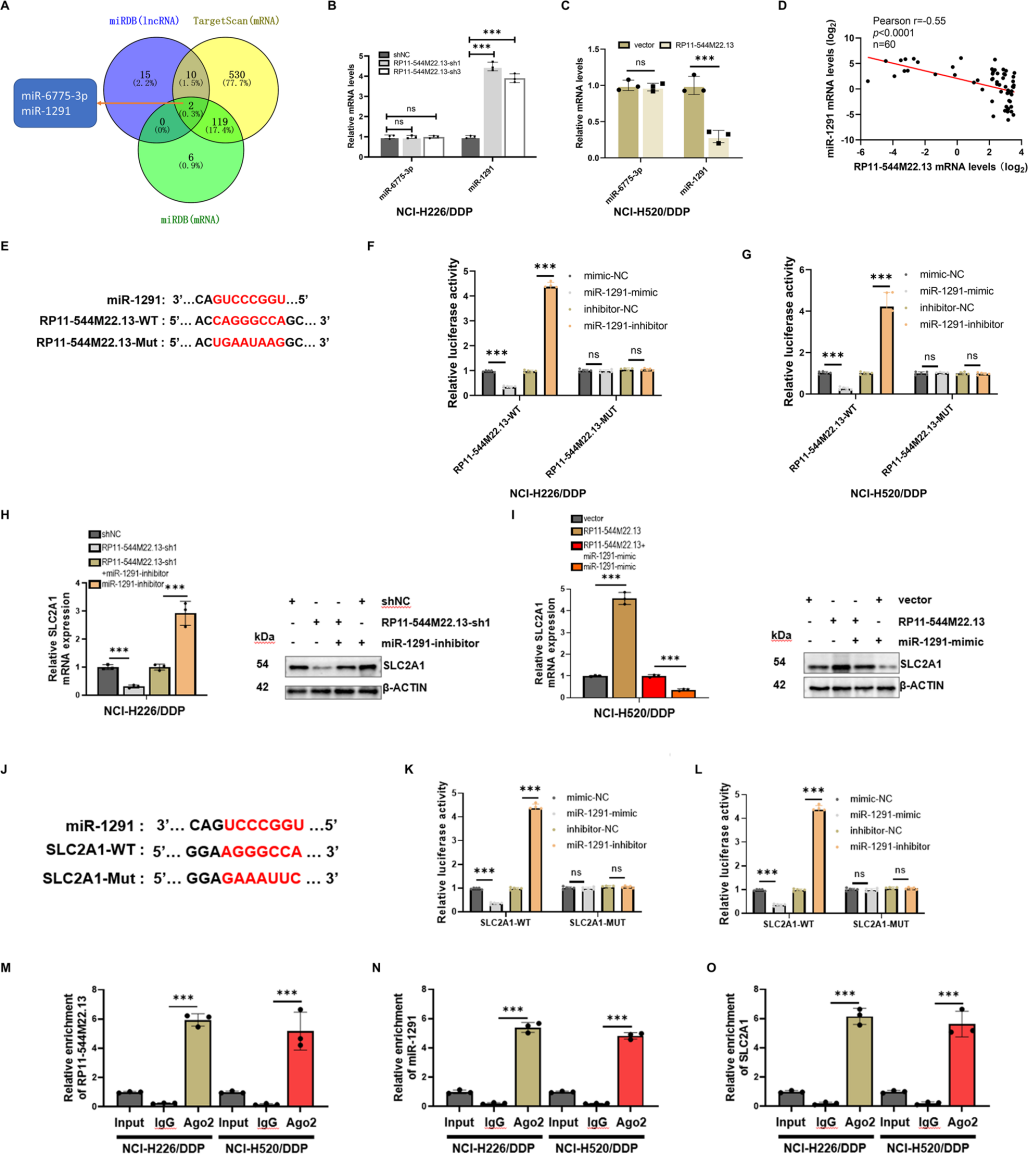
RP11-544M22.13 regulates the expression of SLC2A1 by acting as a sponge for miR-1291. **A** Venn diagram analysis of three databases to identify potential miRNA regulated by RP11-544M22.13. **B**, **C** Manipulation of RP11-544M22.13 expression to examine the level changes of miRNA. **D** Correlation analysis of RP11-544M22.13 and miR-1291 expression in NSCLC tissues. **E** Prediction of potential binding sites between RP11-544M22.13 and miR-1291, and design of mutant plasmids using the miRDB database. **F**, **G** Manipulation of miR-1291 expression to assess the fluorescence activity of RP11-544M22.13. **H** Knockdown of RP11-544M22.13 and miR-1291 expression to examine the mRNA and protein levels of SLC2A1. **I** Overexpression of RP11-544M22.13 and miR-1291 expression to examine the mRNA and protein levels of SLC2A1. **J** Prediction of binding sites between miR-1291 and SLC2A1 and construction of mutant plasmids using the miRDB database. **K**, **L** Manipulation of miR-1291 expression to assess the fluorescence activity of SLC2A1. **M**–**O** RNA immunoprecipitation (RIP) experiments using AGO2 antibody to evaluate the enrichment levels of RP11-544M22.13, miR-1291, and SLC2A1.

Most lncRNAs located in the cytoplasm regulate the expression of downstream target genes through the ceRNA mechanism [35798238]. In order to find out whether RP11-544M22.13 regulates the expression of SLC2A1 through the ceRNA mechanism, we selected miRNAs (miR-6775-3p and miR-1291) (Fig. 6A). Among them, only miR-1291 was transcriptionally regulated by RP11-544M22.13, and when RP11-544M22.13 was knocked down, the expression of miR-1291 was reduced, and vice versa (Fig. 6B, C). Furthermore, RP11-544M22.13 and miR-1291 showed a significant negative correlation in NSCLC tissue samples (Fig. 6D). Then, we designed the RP11-544M22.13-WT and RP11-544M22.13-MUT constructs through the binding sites of RP11-544M22.13 and miR-1291 predicted by miRDB (Fig. 6E). Through luciferase reporter gene experiments, we found that overexpression of miR-1291 significantly reduced the fluorescence activity of RP11-544M22.13-WT construct, and vice versa. However, these changes did not occur in the RP11-544M22.13-MUT construct (Fig. 6F, G).

**Fig. 7:**
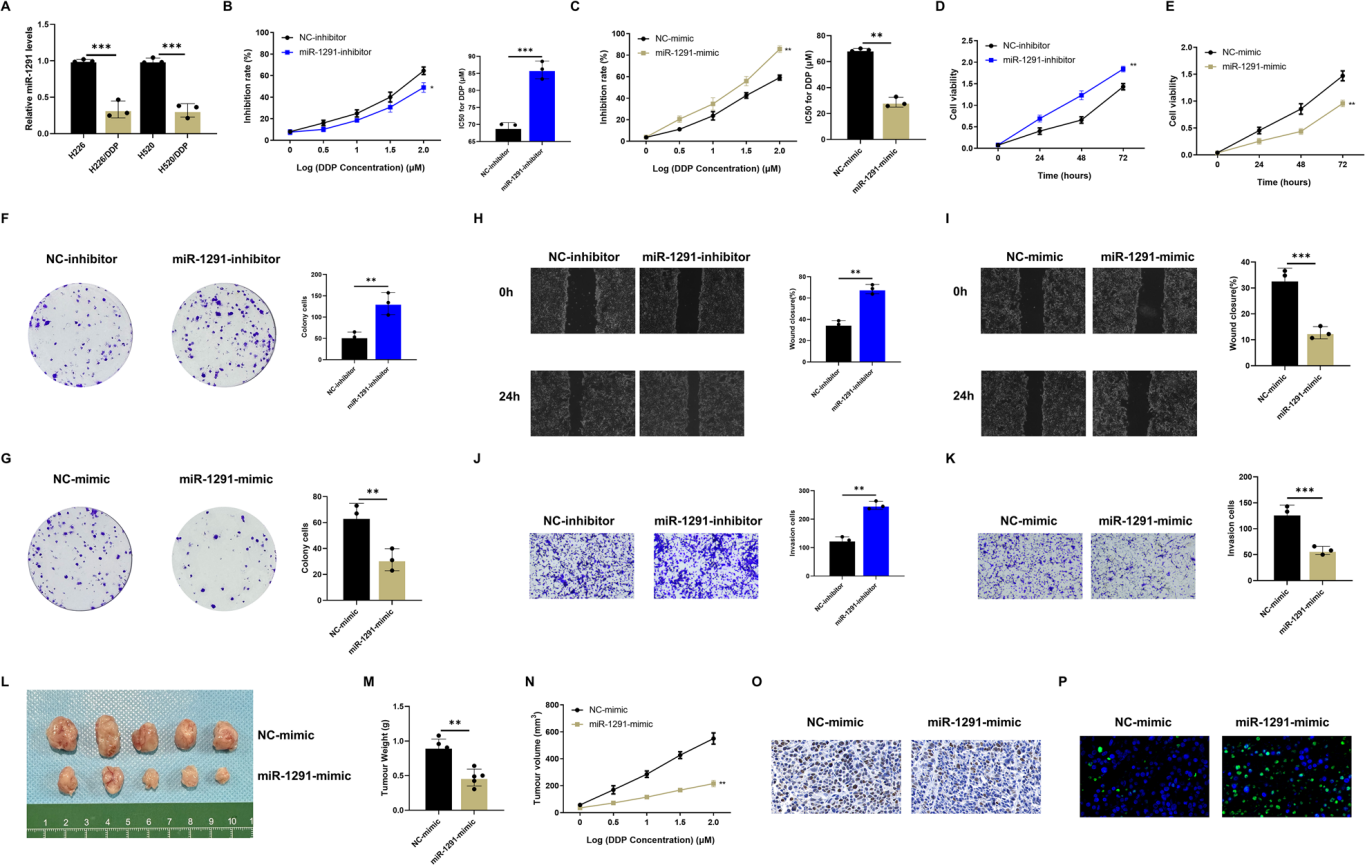
MiR-1291 inhibits the DDP resistance of NSCLC. All NSCLC cells were treated with 1 μg/ml DDP. **A** Expression of miR-1291 in DDP-resistant NSCLC cell lines. **B** Detection of DDP sensitivity of miR-1291 knockdown NSCLC cells (**C**) Detection of DDP sensitivity of miR-1291 overexpression NSCLC cells. **D** Detection of the proliferation ability of miR-1291 knockdown NSCLC cells by CCK8 assay. **E** Detection of the proliferation ability of miR-1291 overexpression NSCLC cells by CCK8 assay. **F** The proliferation ability of miR-1291 knockdown NSCLC cells was detected by colony formation assay. **G** The proliferation ability of miR-1291 overexpressing NSCLC cells was detected by colony formation assay. **H** The migration ability of miR-1291 knockdown NSCLC cells was detected by scratch assay. **I** The migration ability of miR-1291 overexpression NSCLC cells was detected by scratch assay. **J** By Transwell Experiment to detect the invasive ability of miR-1291 knockdown NSCLC cells. **K** Transwell assay to detect the invasive ability of miR-1291 overexpressing NSCLC cells. **L** miR-1291 overexpression NCI-H520 cells were subcutaneously implanted into the left axilla of BALB/c-nu mice for tumor formation experiment. **M** Measure the size of subcutaneous tumors. **N** Measure the growth rate of subcutaneous tumors. **O** KI-67 staining demonstrates the proliferative capacity of tumor cells, 400× magnification. **P** TUNEL staining reveals the apoptotic level of tumor cells, 400× magnification.

We then found that knockdown of miR-1291 could upregulate the expression of SLC2A1, and knockdown of miR-1291 could rescue the inhibitory effect of RP11-544M22.13 knockdown on SLC2A1 expression (Fig. 6H). Overexpression of miR-1291 showed the opposite result (Fig. 7I). We designed the SLC2A1-WT and SLC2A1-MUT constructs through the binding sites of SLC2A1 and miR-1291 predicted by miRDB (Fig. 7J). Through luciferase reporter gene experiments, we found that overexpression of miR-1291 significantly reduced the fluorescence activity of SLC2A1-WT construct, and vice versa. However, these changes did not occur in the SLC2A1-MUT construct (Fig. 7K, L). This was further confirmed by RNA immunoprecipitation (RIP) experiment using an AGO2 antibody specific for miRNA binding (Fig. 7M–O). These data suggest that RP11-544M22.13 acts as a miR-1291 sponge to regulate SLC2A1 expression in NSCLC cells.

### MiR-1291 inhibits the DDP resistance of NSCLC

Since miR-1291 has not been studied in NSCLC. In order to explore the specific function of miR-1291 in NSCLC DDP resistance and verify the reliability of the RP11-544M22.13/miR-1291/SLC2A1 axis, we designed knockdown and overexpression plasmids of miR-1291. The study found that the expression of miR-1291 was significantly reduced in DDP-resistant NSCLC (Fig. 7A), and knockdown of miR-1291 significantly reduced the sensitivity of NSCLC to DDP (Fig. 7B). In addition, CCK8 and clone formation experiments showed that knockdown of miR-1291 significantly reduced the proliferation ability of NSCLC (Fig. 7D–F). Transwell and scratch experiments showed that knockdown of miR-1291 significantly reduced the invasion and migration ability of NSCLC (Fig. 7H–J). In contrast, overexpression of miR-1291 resulted in the opposite results. These experiments suggested that lncRNA MIR-1291 promoted DDP resistance and oncogenic behavior in NSCLC (Fig. 7C, E, G, I, K). In addition, we also verified the function of miR-1291 in a mouse transplant tumor model and found that overexpression of miR-1291 could inhibit the proliferation rate and volume of subcutaneous tumors (Fig. 7L–N). Overexpression of miR-1291 could also significantly reduce the level of ki67 in tumor cells (Fig. 7O). Tunel staining showed that overexpression of miR-1291 could significantly increase the apoptosis level of tumor cells (Fig. 7P). The above experiments proved that miR-1291 inhibits the DDP resistance of NSCLC both in vivo and in vitro.

### The transcription factor USF1 is responsible for the upregulation of RP11-544M22.13 in NSCLC

**Fig. 8:**
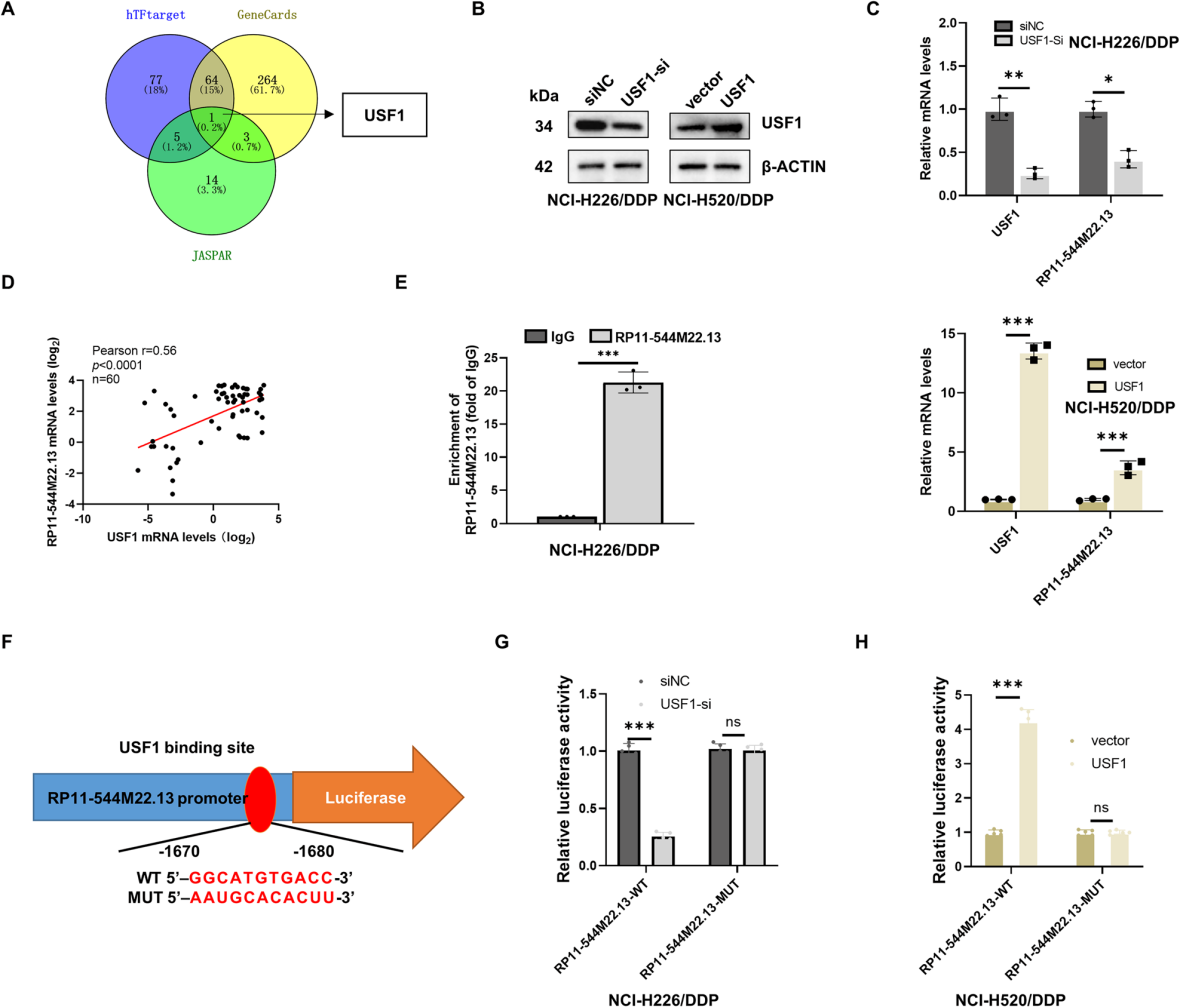
USF1 has been identified as a transcription factor for RP11-544M22.13. **A** A Venn diagram shows three transcription factor databases predicting potential regulators of RP11-544M22.13. **B** Construction of NSCLC cell lines with knockdown and overexpression of USF1. **C** Knockdown or overexpression of USF1 to examine the mRNA expression of RP11-544M22.13. **D** Analysis of the correlation between USF1 and RP11-544M22.13 in NSCLC tissues. **E** CHIP assay demonstrates the binding level of USF1 to RP11-544M22.13. **F** JASPAR database displays potential binding regions of RP11-544M22.13 and USF1, and a luciferase reporter gene plasmid is designed for the promoter region of RP11-544M22.13 based on this information. **G**, **H** Manipulation of USF1 expression to assess the fluorescence activity of the promoter region of RP11-544M22.13.

To clarify the reason for the upregulation of RP11-544M22.13 in NSCLC, we used geneCARDS, hTFtarget and JASPAR databases to predict that USF1 might be the transcription factor regulating the expression of RP11-544M22.13 (Fig. 8A). We then constructed USF1-knockdown NSCLC cell lines and found that USF1 knockdown significantly decreased the expression of RP11-544M22.13, whereas overexpression of USF1 significantly increased the expression of RP11-544M22.13 (Fig. 8B, C). We also found that USF1 was strongly positively correlated with RP11-544M22.13 in NSCLC tissues (Fig. 8D). ChIP qPCR results demonstrated that USF1 directly binds to the RP11-544M22.13 promoter region (Fig. 8E). We constructed a reporter plasmid based on the binding site between USF1 and RP11-544M22.13 promoter region predicted by JASPAR (Fig. 8F). Our results showed that knockdown of USF1 significantly reduced the fluorescence activity in RP11-544M22.13-WT group and vice versa, however these changes were not observed in RP11-544M22.13-MUT group (Fig. 8G, H). The above results indicated that USF1 acts as a transcription factor leading to the upregulation of RP11-544M22.13 in NSCLC.

## Discussion

Despite improvements in diagnostic and therapeutic strategies, the prognosis of NSCLC patients remains unfavorable. Currently, treatment options for NSCLC patients include surgery, radiotherapy, chemotherapy, targeted therapy, and immunotherapy. However, chemoresistance is a huge obstacle.

Because cancer cells multiply and divide at an uncontrolled rate, their energy demands are higher. The Warburg effect that occurs during cancer cell development leads to increased glycolysis, which sustains cell proliferation, doubles the energy supply, and creates the acidic environment required for tumor cell dissemination and invasion [6, 11]. This pathological alteration of energy metabolism results in epigenetic and genetic changes and produces multiple severely impaired phenotypes that further enhance cancer cell proliferation and invasiveness as well as drug resistance [8]. Glycolysis is a complex chain of enzymatic reactions involving transporters for the internalization of glucose into cells as well as a variety of enzymes and metabolites, many of which have been shown to be involved in the induction of drug resistance. As for SLC2A1, as an important glucose transporter, it can regulate glycolysis in NSCLC [12, 13]. And SLC2A1 has been reported to induce chemotherapy resistance in acute myeloid leukemia [14], but its role in drug-resistant NSCLC has not been elucidated. We confirmed for the first time that lncRNA RP11-544M22.13 enhances glycolysis in NSCLC through SLC2A1, thereby leading to DDP resistance in NSCLC.

Previous studies have shown that lncRNAs act as ceRNAs to interact with miRNAs, allowing miRNAs to target gene expression [15]. miR-1291 has become a tumor suppressor in many types of tumors, but its role in NSCLC is still unclear [16]. In this study, we found a negative correlation between miR-1291 and RP11-544M22.13 expression in NSCLC. Overexpression of miR-1291 reduced luciferase activity of RP11-544M22.13 or SLC2A1 reporter genes. Rescue experiments showed that miR-1291 knockdown reduced the inhibitory effect of RP11-544M22.13 on SLC2A1. Our mechanistic studies suggest that RP11-544M22.13 functions as a molecular sponge for miR-1291. In particular, RP11-544M22.13 repressed miR-1291 expression, thereby upregulating the expression of the key glycolysis gene SLC2A1.

In conclusion, we provide evidence that RP11-544M22.13 enhances DDP resistance in NSCLC cells in vitro and in vivo. In addition, RP11-544M22.13 also promoted the proliferation, invasion and metastasis of NSCLC cells. Furthermore, the effect of RP11-544M22.13 on chemotherapy resistance and malignant potential of NSCLC cells was mediated by miR-1291/SLC2A1 signaling, which provided a new target for NSCLC therapy. At the same time, the development of small-molecule drugs targeting RP11-544M22.13 in the future will help combined chemotherapy in the treatment of chemotherapy-resistant advanced NSCLC patients.

## Materials and methods

### Tissue sample

We gathered 60 pairs of NSCLC tissues and corresponding nontumor tissues from NSCLC patients at Renmin Hospital of Wuhan University, China. All patients consented, and none had undergone radiation or chemotherapy before surgery. The study, approved by the Ethics Committee (WDRY2021-K037), followed the Declaration of Helsinki principles.

### Bioinformatics analysis

We utilized GSEA v4.0 software to perform gene enrichment analysis on NSCLC patient data obtained from the TCGA dataset. Table S1 provides URLs for online analytical websites.

### Cell culture

NSCLC cell lines (NCI-H226, NCI-H1703, NCI-H596, NCI-H520, LTEP-s, A549, and CALU3) were acquired from the Chinese Academy of Sciences. Normal lung cells (BEAS-2B) were obtained from Fuxiang Biotechnology Company. All cells were cultured in DMEM with 10% FBS and maintained at 37 °C with 5% CO2.

### qRT-PCR and Western blot

We employed TRIzol for RNA extraction, followed by reverse transcription. qRT-PCR utilized the KiCqStart® SYBR® Green qPCR ReadyMix (KCQS02, Sigma). Primer sequences are provided in Table S2. Nucleocytoplasmic RNA fractionation analysis was conducted using the Nuclei PURE Prep (NUC201, Sigma) to isolate cytoplasmic and nuclear RNAs.

Protein extraction involved the use of RIPA buffer, followed by transfer onto NC membranes. Antibodies listed in Table S3 were then applied to the membranes. ECL signals were detected using a NIKON imaging system.

### Chemotherapy resistance assessment

To assess chemotherapy resistance in NSCLC cells, an MTT assay was performed. Cells were treated with different doses of DDP, incubated with MTT (Sigma) for 4 h, and the resulting optical density at 450 nm was measured [5].

To assess proliferation capacity, NSCLC cells were cultured in a 96-well plate overnight for adherence. Following transfection, MTT (Sigma) was added, and the subsequent steps followed the previous experimental protocol [5].

### Plasmids, siRNA transduction, and shRNA transfection

RP11-544M22.13 overexpressing NSCLC cells were transfected with plasmids, shRNA, and siRNA using Lipo 3000 and pcDNA 3.1-RP11-544M22.13. GenMuteTM (SL100568, SignaGen, USA) was utilized for siRNA delivery. Gene Create Co. (Wuhan, China) packaged lentiviruses containing shRNA or a negative control.

### Xenograft model

Male BALB/c nude mice, aged 6 weeks, were obtained from Wuhan University’s Laboratory Animal Center for a xenograft study [17, 18]. After subcutaneous tumor formation, DDP (5 mg/kg per day) was injected intraperitoneally into mice. Tumor size was measured every 5 days. Tumor and metastasis samples were collected at 30 days and 6 weeks, respectively, following subcutaneous and intravenous injections of NSCLC cells. All animal procedures were conducted in accordance with approved guidelines from the Chinese Animal Welfare Committee (Approval Number: 20191211).

### Oncology behavior

Tests were performed to assess the impact, including cell proliferation, colony formation, CCK-8 assays, wound-healing assay for cell migration, and Transwell invasion assay for quantifying cell invasion.

### Dual luciferase reporter assay

We constructed reporter plasmids by inserting wild-type (WT) and mutant (MUT) sequences of miR-1291, USF1, and the RP11-544M22.13.3 promoter region into the pGL3 basic vector. After Lipofectamine 3000 transfection for 48 h, we measured luciferase activity using the Renilla-Firefly Luciferase Dual Assay Kit (HY-K1013, MCE).

### Chromatin immunoprecipitation (ChIP)

ChIP analysis was performed in NSCLC cells to explore USF1 interactions with the RP11-544M22.13.3 promoter regions. Sonication and formaldehyde crosslinking were utilized in the process, and immunoprecipitation with an anti-USF1 antibody and IgG control was conducted.

### Transcriptomics

RNA integrity was evaluated using an Agilent 2100 system for RNA-seq. Illumina-compatible libraries were prepared with the VAHTS mRNA-seq v2 Library Prep Kit and sequenced on an Illumina NovaSeq platform. Differentially expressed genes meeting the criteria of *p* < 0.05 and |log2 fold change| > 1 were identified.

### Metabolic assays

Glucose and lactate levels were analyzed using kits from Jiancheng Bioengineering Institute, while ATP levels were measured with a kit from the same institute. The Seahorse XFp analyzer determined metabolic flux rate and calculated ECAR (extracellular acidification rate) by culturing cells in a glutamine medium and adjusting temperature and pH prior to analysis.

### Statistical analysis

Statistical analysis was conducted using Prism 7.3 and SPSS 25.0. A two-sided unpaired t-test was used for the xenograft growth experiment, and Pearson correlation analysis was used to assess associations between gene expression levels. A *p* < 0.05 was deemed statistically significant after three iterations of each trial.

## Supplementary information

Supplementary Table S1–S4

Original Data

## Data Availability

The data that support the findings of this study are available from the corresponding author upon reasonable request.
