# Supplementary material for: A novel lncRNA RP11-544M22.13 enhances glycolysis-induced cisplatin resistance in non-small cell lung cancer
Source: Cell Death Discov. 2025 Nov 27;12:24. doi: 10.1038/s41420-025-02873-3 (PMC12808317; doi:10.1038/s41420-025-02873-3)
Supplement: Supplementary file 1 — Supplementary Table S1–S4 [file 41420_2025_2873_MOESM1_ESM.docx]

**Supplementary Table S1** Online analysis website website URL

GEPIA <http://gepia.cancer-pku.cn/>

UCSC <http://genome.ucsc.edu/>

Mirdb <http://mirdb.org/>

LncBase [http://carolina.imis.athena-innovation.gr/diana_tools/](http://carolina.imis.athena-innovation.gr/diana_tools/%20) Starbase <http://starbase.sysu.edu.cn/starbase2/index.php>

Targetscan <http://www.targetscan.org/vert_72/>

TCGA-LIHC [https://portal.gdc.cancer.gov/](https://portal.gdc.cancer.gov/%20)

**Supplementary Table S2** Primer sequences, siRNAs and shRNA used in this study.

| Primer name |  | Sequence (5’-3’) |
| --- | --- | --- |
| RP11-544M22.13 | F | TCTGGCCCCTGATGTCTGTA |
|  | R | AAGAGCCCAGAACCAAGCTC |
| miR-1291 | F | TCGTACATACTGCTGAGCTAG |
|  | RT | GTCGTATCCAGTGCAGGGTCCGAGGTATTCGCACTGGATACGACACTGCT |
| CDK1 | F | CCATACCCATTGACTAACTAT |
|  | R | ACCCCTTCCTCTTCACTTTC |
| ALDOA | F | CATTCTGGCTGCGGATGAGTCT |
|  | R | CACACGGTCATCAGCACTGAAC |
| BPNT1 | F | CACTGTGTTGATGCGGTTGG |
|  | R | GTGCCAATCGGTCAGCTTTG |
| ENO2 | F | CGTTACTTAGGCAAAGGTGTCC |
|  | R | CTCCAGCATCAGGTTGTCCAGT |
| SLC2A1 | F | GGCCAAGAGTGTGCTAAAGAA |
|  | R | ACAGCGTTGATGCCAGACAG |
| GALK1 | F | TGTCCAGCTCAGCATCCTTG |
|  | R | TGACACCCAAGCATACACCC |
| PLOD1 | F | CGCCATGGATCTGTGGACCTGTT |
|  | R | CGGGTCCACTTATGGCATCCGAG |
| MERTK | F | CGAGCTCGGATCTCTGTTCA |
|  | R | GAGGGGGCATAATCTACCCA |
| SDHC | F | CTCGGCCTCCCAAAGAGCTGAGATTA |
|  | R | CTCATCTACATAGCAGTATTTTGGTTGAGTAA |
| VEGFA | F | CACCGAAGGAGACAGTGAATCC |
|  | R | GCTGTTCTGGAGTAAGCTTGTGC |
| USF1 | F | TCCCAGACTGCTCTATGGAGA |
|  | R | CGGTGGTTACTCTGCCGAAG |
| RP11-544M22.13-sh1 |  | UUUGGCUUCAAGUUCUCUGUU |
| RP11-544M22.13-sh2 |  | UAUUCAAAGAUCCCUGCCAUU |
| RP11-544M22.13-sh3 |  | UUGGCUUCAAGUUCUCUGAUU |
| USF1-si |  | AAGUAAGUAUAGUGCGUCU |
| SLC2A1-sh |  | AGUCUCAGGAACUUUGAAGUU |

**Supplementary Table S3 Primary antibodies used in this study**

| Antigens | Manufacturer | Catalog Number | Application |
| --- | --- | --- | --- |
| SLC2A1 | Cell Signaling Technology | #73015 | 1:1000 for WB; |
| USF1 | Abcam | ab180717 | 1:200 for WB; |
| β-actin | Abcam | ab8227 | 1:1000 for WB; |
| IgG | Servicebio | GB23303 | IP |
|  |  |  |  |

**Supplementary Table S4** Correlation between clinicopathological features and RP11-544M22.13 expression in NSCLC tumor tissues.

| Characteristics | Number | RP11-544M22.13 expression | | P value |
| --- | --- | --- | --- | --- |
|  |  | High | Low |  |
| Gender |  |  |  | *0.604* |
| Male | 32 | 17 | 15 |  |
| Female | 28 | 13 | 15 |  |
| Age |  |  |  | *0.787* |
| ＜65 | 21 | 10 | 11 |  |
| ≥65 | 39 | 20 | 19 |  |
| Tumor size |  |  |  | ***0.005^*^*** |
| ≤5cm | 23 | 4 | 16 |  |
| ＞5cm | 37 | 26 | 11 |  |
| TNM staging |  |  |  | ***0.001^*^*** |
| I-II | 15 | 2 | 13 |  |
| III-IV | 45 | 28 | 17 |  |
| Lymph node metastasis |  |  |  | ***0.002^*^*** |
| Absent | 34 | 11 | 23 |  |
| Present | 26 | 19 | 7 |  |

*The expression of RP11-544M22.13 were compared between the tumor tissue and the normal tissue. Bold italics indicate statistically significant values. **p*<0.05
