# Supplementary figures and images for: A novel lncRNA RP11-544M22.13 enhances glycolysis-induced cisplatin resistance in non-small cell lung cancer
Source: Cell Death Discov. 2025 Nov 27;12:24. doi: 10.1038/s41420-025-02873-3 (PMC12808317; doi:10.1038/s41420-025-02873-3)

Figure 3N

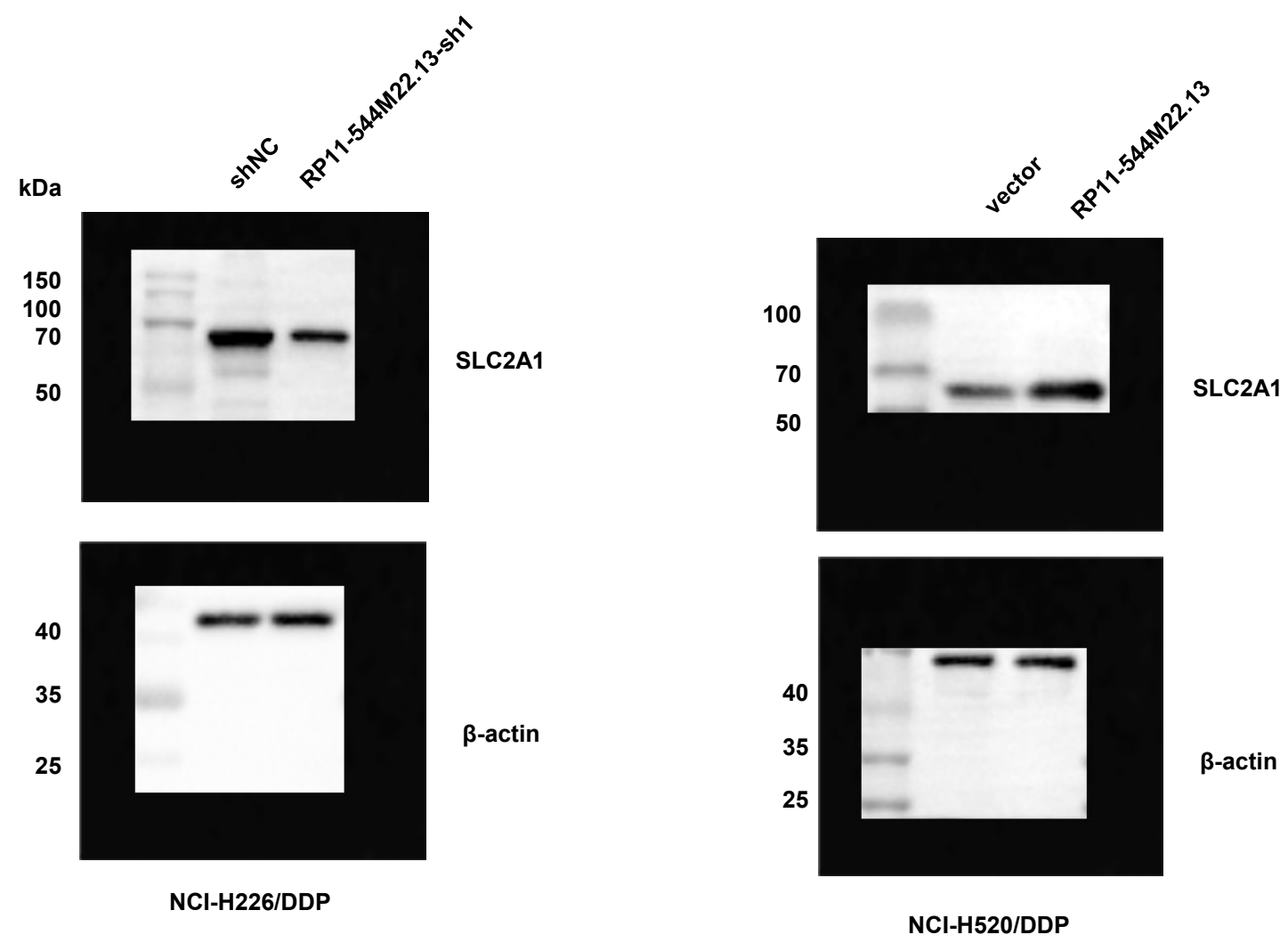

Figure 6H

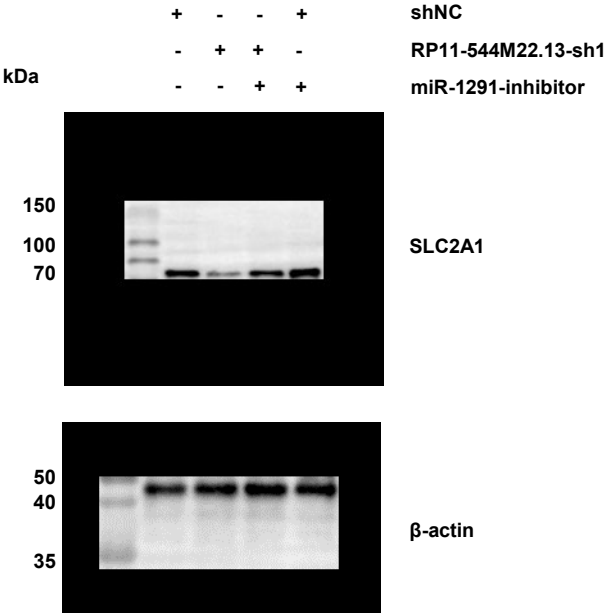

Figure 6I

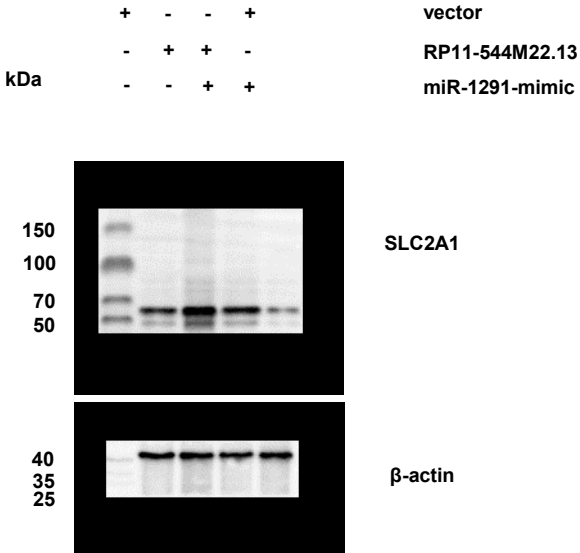

Figure 8B

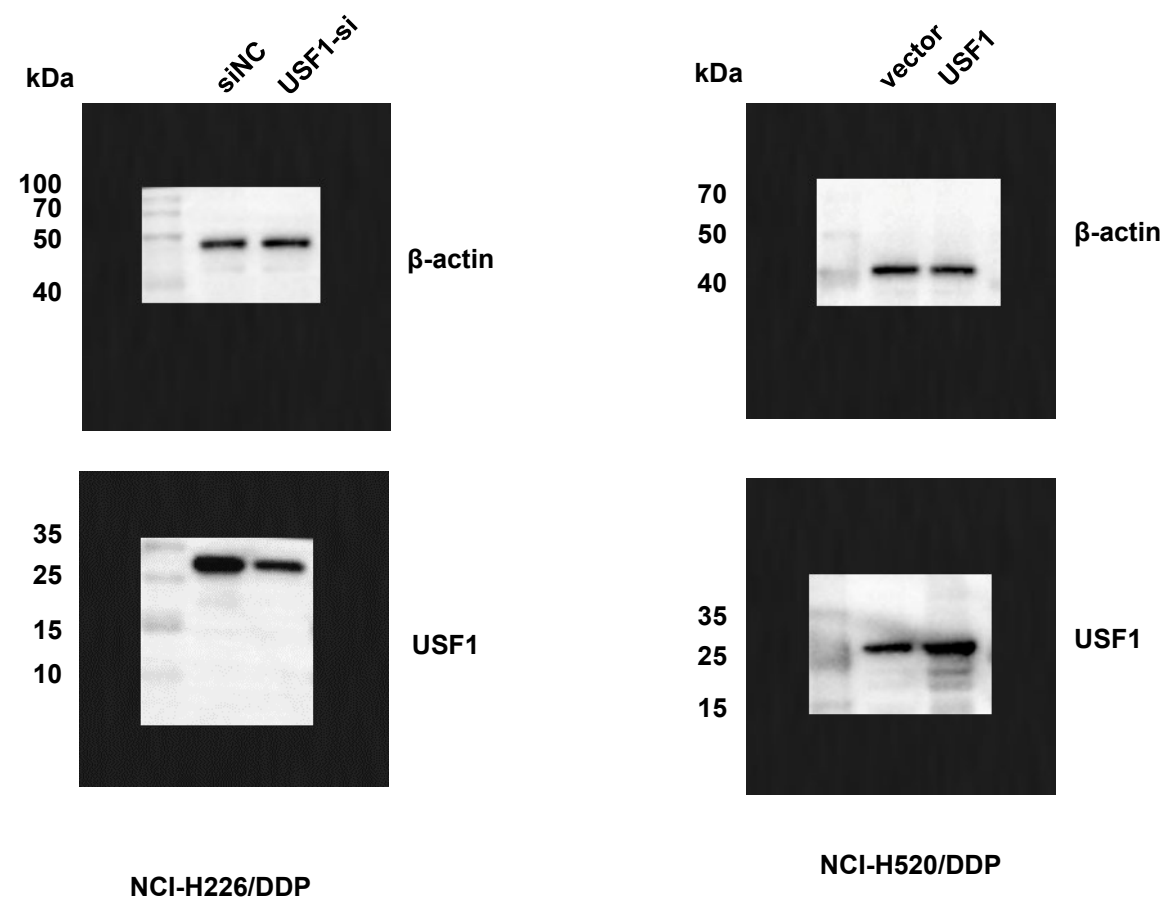

Supplement: Supplementary file 2 — Original Data [file 41420_2025_2873_MOESM2_ESM.pdf]
